# Use of Consumer Wearable Devices to Promote Physical Activity: A Review of Health Intervention Studies

**DOI:** 10.15436/2378-6841.16.1123

**Published:** 2016-11-30

**Authors:** Steven S. Coughlin, Jessica Stewart

**Affiliations:** Department of Clinical and Digital Health Sciences, College of Allied Health Sciences, Augusta University, Augusta, GA

**Keywords:** Monitoring, Obesity, Physical activity, Randomized controlled trials, Smartphone applications, Weight loss

## Abstract

**Background:**

Although many wearable devices for monitoring and tracking physical activity are available to consumers, relatively few research studies have been conducted to determine their efficacy in promoting health.

**Methods:**

In this article, data on the use of consumer wearable devices in promoting healthy behaviors are summarized based upon bibliographic searches in PubMed and Psychology and Behavioral Sciences Collection with relevant search terms through September 2016.

**Results:**

A total of 274 articles were identified in the bibliographic searches. By screening abstracts or full-text articles, six pre/post test trials and seven randomized controlled trials were identified. In initial trials, consumer wearable devices have been shown to increase physical activity and help users lose weight. However, the number of studies completed to date is small and limited by small sample sizes, short study durations, and uncertain generalizability of the findings.

**Conclusions:**

Future studies should utilize randomized controlled trial research designs, larger sample sizes, and longer study periods to better establish the efficacy of wearable devices in promoting physical activity. Additional research is needed to determine the feasibility and effectiveness of wearable devices in promoting physical activity and weight loss in community settings including communities affected by health disparities. Studies focusing on children and adolescents are also needed.

## Introduction

Sedentary behavior and physical inactivity are important public health issues (Rabin, et al., 2011; Vansaun, 2013). Approximately one-third of adults in the U.S. are physically inactive (Rabin, et al., 2011). The increasing prevalence of obesity in the U.S. and many other countries and the independent association of obesity with several forms of cancer, cardiovascular disease, diabetes and other forms of chronic illness have prompted interest in identifying efficacious ways to promote physical activity and reduce obesity (Vansaun, 2013). Among cancer survivors and persons living with other chronic illnesses, maintaining a healthy body weight reduces the risk of disease recurrence or progression (Thompson, et al., 2012).

In the U.S., approximately 35% of adults and 17% of youths are obese (Johnson, et al., 2014; Dietz, 2015). Based on data from the 2013 BRFSS survey, only half of U.S. adults (50.2%) met guidelines for physical activity and an additional 11.7% only partially met the guidelines. Wearable devices overcome some limitations of traditional in-person programs for physical activity and weight management programs. Established interventions for physical activity and weight loss are resource-intensive and time-consuming, factors that limit full participation and widespread dissemination. Wearable devices that monitor physical activity are less expensive than a gym membership or many types of exercise equipment (Hartman, et al., 2015).

Rapid advances have occurred in relatively low-cost wearable devices that assist consumers to monitor their physical activity and become more active (Hartman, et al., 2015; Wang, et al., 2015; Cadmus-Bertram, et al., 2015; Cadmus-Bertram, et al., 2015a; Bade, et al., 2015; Jordan, et al., 2015; Quintiliani, et al., 2016; Hartman, et al., 2016). Devices such as the Fitbit and Jawbone have the ability to measure a variety of activity-related outcomes including steps, distance, heart rate, active minutes, calories, and sleep. Additionally, users can access the app and web interface to socialize with friends and complete group challenges. Fitbit devices have shown high validity and reliability (ICC 0.71 - 1.00) (Noah, et al., 2013; Diaz, et al., 2015; Evenson, et al., 2015) and a growing amount of research has successfully incorporated Fitbit devices into technology-oriented lifestyle interventions to increase physical activity, reduce overweight/obesity, and manage chronic conditions (24 - 31). Users can track minutes of physical activity, steps per day, and floors climbed per day enabling them to receive feedback on their activity.

This article provides a review of published studies on the acceptability and efficacy of wearable devices to promote physical activity and weight loss. Of particular interest were randomized controlled trials of the efficacy of consumer wearable devices to promote physical activity and weight loss. Studies of the reliability and validity of wearable devices for tracking physical activity were recently systematically reviewed by Evenson, et al., (2015) and were not considered in the current review. This review also does not consider wearable devices and systems that have been used to monitor activity in clinical studies of patients recovering from surgery or receiving rehabilitation or treatment for chronic diseases such as osteoarthritis, chronic heart failure, diabetes, peripheral neuropathy, or chronic obstructive pulmonary disease (Benzo, 2009; Allet, et al., 2010; Patel, et al., 2012; Cook, et al., 2013; Chiauzzi, et al., 2015). In addition, studies that employed research devices not intended for routine use by consumers (e.g., research grade accelerometers) were beyond the scope of this review.

## Materials and Methods

The present review is based upon bibliographic searches in PubMed and Psychology and Behavioral Sciences Collection (PBSC) and relevant search terms. Articles published in English from 1993 through September 2016 were identified using the following MeSH search terms and Boolean algebra commands. The following search terms and commands were used: (physical activity and ((Fitbit) or ((Jawbone) and (monitoring)) or wearable device). The searches were not limited to words appearing in the title of an article. Information obtained from bibliographic searches (title and topic of article, information in abstract, geographic locality of a study, and key words) was used to determine whether to retain each article identified in this way. In addition, the references of published articles were reviewed. Studies of the reliability and validity of wearable devices for monitoring physical activity were excluded along with those that employed research technologies not intended for routine use by consumers.

## Results

A total of 274 article citations were identified in the bibliographic searches. After screening the abstracts or full texts of these articles, six pre/post-test trials and seven randomized controlled trials of the efficacy and acceptability of consumer wearable devices to promote physical activity or help manage weight were identified.

Kurti and Dallery, (2013), conducted a non randomized trial of a Fitbit-based physical activity intervention among 12 sedentary adults > 50 years of age. Across participants, steps increased 182% from screening to the end of the intervention when a monetary incentive was provided, and 108% when no monetary incentive was offered.

Washington et al., (2014), conducted a nonrandomized trial of a Fitbit-based physical activity intervention among 11 healthy college students (6 women, 5 men). Participants increased overall step counts 23% overall (p = 0.039).

Martin et al., (2015), conducted a randomized trial of a FitBug and physical activity text messaging intervention delivered via smartphones. The participants were 48 ouptatients (46% women; 21% nonwhite; mean age 58 years) in Baltimore, MD. Participants receiving texts increased their daily steps over those not receiving texts by 2,534 (P < 0.0001) and over controls blinded to activity data through a smartphone access by 3,376 (P < 0.001).

Hayes and Van Camp, (2015), piloted a Fitbit-based physical activity intervention for children that occurred over 22 sessions. The goal was to increase physical activity during recess. Six 8-year old girls from a 3, grade classroom in Wilmington, NC participated in the study. Steps taken during the intervention period were 47% higher than at baseline. In addition, the percentage of recess spent in moderate-to-vigorous physical activity was 25% higher during the intervention.

Yingling, et al., (2016), conducted a two-week pilot study of a physical activity monitoring device (Dynamo Activity Tracker) and focus groups as part of community-based participatory research. Participant wristbands recorded data on 10.1 + 1.6 days; two participants logged cardiovascular health factors on the website. Focus group transcripts revealed that participants felt positively about incorporating the device in their church-based populations, after improvements were made in device training, hub accessibility, and device feedback.

Naslund, et al., (2016), conducted a non-randomized study of a 6-month intervention in which Fitbit devices and smartphone devices were provided. The study participants were people with serious mental illness and obesity. The participants, who wore Fitbits for an average of 84.7 % of the days enrolled in the study, were highly satisfied with the devices. Some participants experienced challenges using the companion mobile application on the smartphone. Cadmus-Bertram, et al., (2015), conducted a randomized controlled trial of a 16-week Fitbit-based physical activity intervention. Women randomized to the control group received a pedometer. Fifty-one postmenopausal women with a body mass index (BMI) > 25.0 kg/m^2^ were included. Relative to baseline, the web-based tracking group increased moderate-to-vigorous physical activity by 29 + 3.5 kg/m^2^ and steps by 789 + 1,979 (p = 0.01), compared to non significant increases in the pedometer group. The web-based tracking group wore the tracker on 95% of intervention days, 96% reported liking the website, and 100% liked the tracker.

Hartman, et al., (2016), conducted a randomized controlled trial of an intervention consisting of use of the Fitbit One to monitor physical activity, the My Fitness Pal smartphone app and website to monitor diet, and coaching calls with trained counselors. The 54 participants were primarily non-Hispanic white, well-educated women with a BMI > 27.5 kg/m^2^ and elevated breast cancer risk, recruited from a mammography clinic in San Diego, CA. At 6-months, intervention participants had lost more weight (4.4 kg vs. 08 kg, p = 0.004) than usual care participants.

Wang, et al., (2016), conducted a randomized controlled trial of a 6-week Fitbit-based physical activity intervention. Participants randomized to the intervention group received both the Fitbit device and three daily short message service text messages. Those randomized to the comparison group received only the Fitbit device. The participants were 67 overweight and obese adults. A significant within-group increase of 4.3 minutes per week of moderate to vigorous intensity physical activity was observed in the comparison group (Fitbit only) (p = 0.04), but no differences in physical activity levels were seen across study groups.

Ashe, et al., (2015), conducted a randomized controlled trial of a 6-month Fitbit-based physical activity intervention. The participants were 25 women ages 55 - 70 years in Vancouver, BC. Controlling for baseline values, the intervention group had an average of 2,080 more steps per day at 6 months compared with the control group. There was an average between group differences in weight loss of -4.3 kg in favor of the intervention.

Choi, et al., (2016), conducted a randomized controlled trial comparing use of Fitbit and smartphone app vs. Fitbit alone to promote physical activity. Thirty pregnant women between 10 - 20 weeks of gestation were included in the trial. At 12 weeks, intervention group participants had a 1,096 step increase in daily steps compared to an increase of 259 steps in controls (p = 0.38). The intervention group reported lower perceived barrier to being active, lack of energy, than the control group at 12 weeks (p = 0.02).

Chung, et al., (2016), conducted a randomized controlled trial comparing use of Fitbit and smartphone app vs. Fitbit alone to promote physical activity. The participants were young adults in North Carolina. One-day challenges were successful in increasing steps. Compliance with daily Fitbit wear was high (73 - 99%).

Jakicic, et al., (2016), conducted a randomized clinical trial with an IDEA design (Innovative Approaches to Diet, Exercise and Activity) where they compared a standard behavioral weight loss intervention (SBWI) (n = 233) and a technology-enhances weight loss intervention (EWLI) (n = 237). The EWLI groups were equipped with commercially available wearable devices, which included web interface technology. The participants were young adults' ages 18 to 35 years in Pittsburgh, PA. There were no significant differences between groups at 24 months.

## Discussion

The number of trials of the effectiveness of consumer wearable devices in promoting physical activity and controlling weight completed to date is small and limited by small sample sizes, short study durations, and uncertain generalizability of findings. Differences in study design (e.g., choice of a comparison group, outcome measures) and wearable device functionalities also increase the difficulty of drawing firm conclusions about their effectiveness in increasing physical activity or helping people to lose weight. Only a handful of studies have focused on children or young adults (Hayes, et al., 2015; Chung, et al., 2016). Nevertheless, the results of this review indicate that it is feasible to use consumer wearable devices to promote physical activity.

Wearable devices offer a useful approach for monitoring physical activity in both clinical research involving patient populations and community-based research that addresses health disparities in at-risk communities (Yingling, et al., 2016). A pilot study employed a wearable device in community-based participatory research involving African Americans at increased risk of cardiovascular disease (Yingling, et al., 2016). Preliminary results indicate that mHealth technology is feasible for physical activity interventions in resource-limited communities (Yingling, et al., 2016). In order for wearable devices to be useful for promoting physical activity and weight loss in communities affected by disparities in obesity and other chronic conditions, the information they provide must be understandable to people with varying levels of health literacy and numeracy (Chiauzzi, et al., 2015).

A variety of wearable devices for monitoring and tracking physical activity are available to consumers, although relatively few have been tested in order to determine their acceptability, usefulness, efficacy or effectiveness in promoting health. An estimated 32 million wearable physical activity trackers will be sold by the end of 2016 and it is projected that sales of these devices will surpass 82 million by 2019 (Parks Associates, 2015; Allied Market Research, 2015). Nevertheless, the uptake of smartphones is much higher in the U.S. population than the use of wearable devices.

## Conclusion

Additional research is needed to examine the efficacy of wearable devices in promoting physical activity and weight loss. Future studies should utilize randomized controlled trial research designs, larger sample sizes, and longer study periods to better explore the intervention capabilities of wearable devices for promoting physical activity. Studies focusing on children and adolescents are also needed.

## Figures and Tables

**Figure 1:**
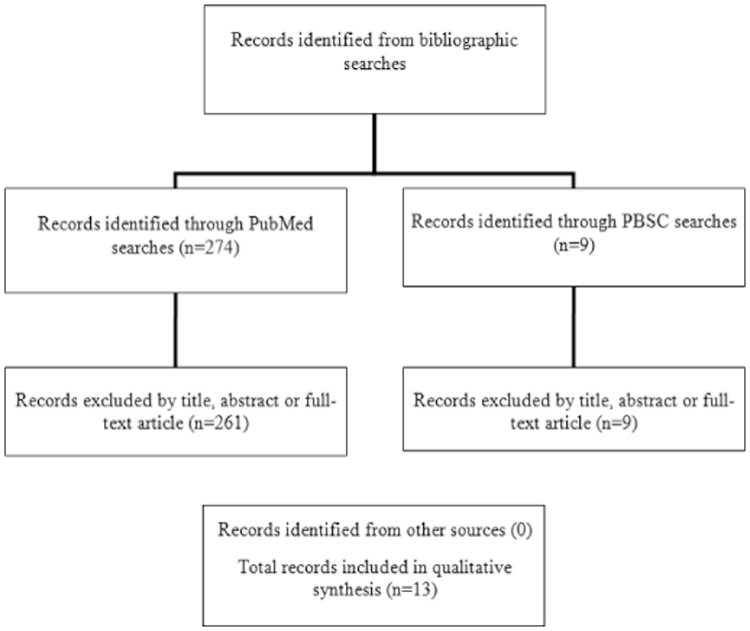
Summary of search and exclusion process: (physical activity and ((Fitbit) or ((Jawbone) and (tracking)) or wearable device)). (physical activity) AND (((fitbit) OR ((jawbone) and (tracking)) OR (wearable device))).

**Table 1:** Trials of consumer wearable devices for promoting physical activity and weight loss (in order of publication date).

Study	Sample	Design	Intervention Period	Results	Limitations
Kurti and Dallery, (2013)	12 sedentary adults > 50 years of age in Gainsville, FL	Nonrandomized trial of a Fitbit-based physical activity intervention.	2 months	Across participants, steps increased 182% from screening to the end of the intervention when a monetary incentive was provided, and 108% when no monetary incentive was offered.	Small sample size, uncertain generalizability, lack of randomized controlled design.
Washington et al. (2014)	11 healthy adults (6 women, 5 men) 18-26 years old in Wilmington, NC	Nonrandomized trial of a Fitbit-based physical activity intervention.	3 weeks	Participants increased overall step counts 23% overall (p = 0.039).	Small sample size, uncertain generalizability, lack of a randomized controlled design.
Martin et al. (2015)	48 outpatients (46% women; 21% non-white; mean age 58 years) in Baltimore, MD	Randomized trial of FitBug and physical activity text messaging intervention delivered via smartphones.	5 weeks	Participants receiving texts increased their daily steps over those not receiving texts by 2,534 (P < 0.0001) and over controls blinded to activity data through a smartphone access (3,376 (P < 0.001).	Small sample size, uncertain generalizability.
Hayes and Van Camp, (2015)	6 girls (8 years old) from a 3rd grade classroom in Wilmington, NC	Fitbit-based physical activity intervention with 22 sessions.	22 sessions, 1- 4× per week.	Steps taken during the intervention period were 47% higher than at baseline, and the percentage of recess spent in moderate-to-vigorous physical activity was 25% higher during the intervention.	Small sample size, uncertain generalizability, nonrandomized design.
Cadmus-Bertram et al. (2015)	Postmenopausal women (n = 51, average age 60 years) with BMI > 25.0 kg/m^2^ (n = 51) in San Diego, CA	Randomized controlled trial of a 16-week Fitbit-based physical activity intervention. Women randomized to the control group received a pedometer.	16 weeks	Relative to baseline, the web-based tracking group increased moderate-to-vigorous physical activity by 29 + 3.5 kg/m^2^ and steps by 789 + 1,979 (p = 0.01), compared to non significant increases in the pedometer group. The web-based tracking group wore the tracker on 95% of intervention days, 96% reported liking the website, and 100% liked the tracker.	Small sample size, uncertain generalizability
Naslund et al. (2016)	People with serious mental illness and obesity (n =11; 73% female; average age 48.2 years; 100% white; average BMI 41.5 kg/m^2^) in Lebanon, NH	Non-randomized study of a 6-month intervention in which Fitbit devices and smartphone devices were provided.	6 months	The participants wore Fitbits for an average of 84.7% of the days enrolled in the study. Participants were highly satisfied with the devices. Some participants experienced challenges using the companion mobile application on the smartphone.	Small sample size, uncertain generalizability, limited availability of quantitative data on participants' use of the Fitbit devices and whether they achieved their steps goals.
Hartman et al. (2016)	Primarily non-Hispanic white, well-educated women (n = 54) with a BMI > 27.5 kg/m^2^ and elevated breast cancer risk, recruited from a mammography clinic in San Diego, CA	Randomized controlled trial of an intervention consisting of use of the Fitbit One to monitor physical activity, the My Fitness Pal smart-phone app and website to monitor diet, and coaching calls with trained counselors.	6 months	At 6-months, intervention participants had lost more weight (4.4 kg vs. 08 kg, p = 0.004) than usual care participants.	Small sample size, uncertain generalizability of results, lack of adherence data regarding use of My Fitness Pal.
Yingling et al. (2016)	African American church members (n = 8; 5 males, 3 females, ages 28-70 years)) in Washington, DC	Two-week piloting of physical activity monitoring device (Dynamo Activity Tracker) and focus group conducted as part of community-based participatory research.	2 weeks	Participant wristbands recorded data on 10.1 + 1.6 days; two participants logged cardiovascular health factors on the website. Focus group transcripts revealed that participants felt positively about incorporating the device in their church-based populations, after improvements were made in device training, hub accessibility, and device feedback.	Small sample size, uncertain generalizability. The short duration of the pilot study limited testing of participant adherence, engagement, retention, and attrition.
Wang et al. (2016)	67 overweight and obese adults (91% female, 61% college graduates, 67% non-Hispanic white, mean age 48.2 years) in San Diego, CA	Randomized controlled trial of a 6-week a Fitbit-based physical activity intervention. Participants randomized to the intervention group received both the Fit-bit device and three daily short message service text messages. Those randomized to the comparison group received only the Fit-bit device.	6 weeks	A significant within-group increase of 4.3 minutes per week of moderate to vigorous intensity physical activity was observed in the comparison group (Fitbit only) (p = 0.04), but no study group differences in physical activity levels were seen.	Small sample size, uncertain generalizability, short duration of the study.
Ashe et al. (2015)	25 women ages 55-70 years in Vancouver, BC, Canada.	Randomized controlled trial of a 6-month Fitbit-based physical activity intervention. The intervention was grounded in the social ecological model.	6 months	Controlling for baseline values, the intervention group had an average of 2,080 more steps per day at 6 months compared with the control group. There was an average between group differences in weight loss of -4.3 kg in favor of the intervention.	Small sample size, uncertain generalizability.
Choi et al. (2016)	Pregnant women (n = 30) between 10 - 20 weeks of gestation in San Francisco, CA.	Randomized controlled trial comparing use of Fitbit and smartphone app vs. Fitbit alone to promote physical activity.	12 weeks	At 12 weeks, intervention group participants had a 1,096 step increase in daily steps compared to an increase of 259 steps in controls (p = 0.38). The intervention group reported lower perceived barrier to being active, lack of energy, than the control group at 12 weeks (p = 0.02).	Small sample size, uncertain generalizability.
Chung et al. (2016)	Young adults in NC.	Nonrandomized trial of a 2-month Fit-bit-based intervention that used Twitter and gamification to promote physical activity and healthy diet.	2 months	One-day challenges were successful in increasing steps. Compliance with daily Fitbit wear was high (73 - 99%).	Nonrandomized design, uncertain generalizability, short duration of intervention.
Jakicic et al. (2016)	470 (233 SBWI, 237 EWLI) adults ages 18 to 35 in Pittsburgh, PA	Randomized trial between standard behavioral (SBWI) and technology-enhanced (EWLI) weight loss interventions	24 months	Weight change at 24 months differed significantly by intervention group (difference, 2.4 kg [95%CI, 1.0 - 3.7]; P = .002). Both groups had significant improvements in body composition, fitness, physical activity, and diet, with no significant difference between groups.	Sample limited to young adults (18 - 35). Device worn on upper arm, not reflect effectiveness of write-worn. Self-reported dietary intake.
